# Osteoconductive Microarchitecture of Bone Substitutes for Bone Regeneration Revisited

**DOI:** 10.3389/fphys.2018.00960

**Published:** 2018-07-19

**Authors:** Chafik Ghayor, Franz E. Weber

**Affiliations:** ^1^Oral Biotechnology and Bioengineering, Department of Cranio-Maxillofacial and Oral Surgery, Center for Dental Medicine, University of Zurich, Zurich, Switzerland; ^2^Zurich Center for Integrative Human Physiology, University of Zurich, Zurich, Switzerland; ^3^Center for Applied Biotechnology and Molecular Medicine, University of Zurich, Zurich, Switzerland

**Keywords:** osteoconduction, pore, bone substitute material, additive manufacturing, lithography, micro architecture, bone regeneration, scaffold

## Abstract

In the last three decades, all efforts in bone tissue engineering were driven by the dogma that the ideal pore size in bone substitutes lies between 0.3 and 0.5 mm in diameter. Newly developed additive manufacturing methodologies for ceramics facilitate the total control over pore size, pore distribution, bottleneck size, and bottleneck distribution. Therefore, this appears to be the method of choice with which to test the aforementioned characteristics of an ideal bone substitute. To this end, we produced a library of 15 scaffolds with diverse defined pore/bottleneck dimensions and distributions, tested them *in vivo* in a calvarial bone defect model in rabbits, and assessed the clinically most relevant parameters: defect bridging and bony regenerated area. Our *in vivo* data revealed that the ideal pore/bottleneck dimension for bone substitutes is in the range of 0.7–1.2 mm, and appears therefore to be twofold to fourfold more extended than previously thought. Pore/bottleneck dimensions of 1.5 and 1.7 mm perform significantly worse and appear unsuitable in bone substitutes. Thus, our results set the ideal range of pore/bottleneck dimensions and are likely to have a significant impact on the microarchitectural design of future bone substitutes for use in orthopedic, trauma, cranio-maxillofacial and oral surgery.

## Introduction

Bones have the natural ability to heal due to the presence of osteoinductive proteins in the bone matrix (Urist, 1965) and mesenchymal progenitor and stem cells in the bone marrow, the periost and other surrounding tissues (Owen and Friedenstein, 1988; Bianco et al., 2008). If bone defects surpass a critical size, however, transplantation of autologous tissue is often a necessary requirement to aid the regenerative process. Bone is the second most frequently transplanted tissue in Europe after blood, with around one million procedures performed annually. The worldwide market of bone replacement material is currently estimated at five billion € and is increasing by 10% every year (Medical Press, 2017). Importantly, the outcome of bone transplantation is not only dependent on the osteoinductive nature of the implanted material, but also on its osteoconductive properties.

Osteoconduction defines a three dimensional process observed when porous structures are implanted in or adjacent to bone. The porous spaces are initially infiltrated by capillaries, perivascular tissues, and osteoprogenitor cells, followed by incorporation of the porous structure within the newly formed bone (Cornell and Lane, 1998). Osteoconduction was first described by Barth (1893) following the histological analysis of the fate of transplanted autologous bone. Findings revealed that the transplanted bone was degraded and replaced by newly formed bone through a process termed creeping substitution (Axhausen, 1909). It has since been demonstrated that during the latter phases of this process, the degradation of the autologous bone liberates calcium phosphates and osteoinductive proteins (Cornell and Lane, 1998), which further serve to enhance bone regeneration Although considered the gold standard for bone repair, the harvesting of autologous bone carries with it the risk of donor site morbidity. This, together with limitations in its availability, have led investigators to seek out alternative bone substitutes, with the aim to developing off-the-shelf products for treating bone defects (Burchardt, 1983). In designing bone substitute, great emphasis is placed on material type, porosity and surface, all of which influence the efficiency of bone ingrowth. The influence of material surface in particular has been a primary focus in the development of dental implants where efficient osseointegration is critical (Buser et al., 1991). For titanium implants, the best surface appeared to be a moderately rough one (Schwartz et al., 1999; Wennerberg and Albrektsson, 2010), whereas for calcium phosphate-based bone substitutes, submicron surface structures outperformed larger micron scaled surfaces in terms of their ability to stimulate the osteogenic differentiation of multipotent stromal cells (Zhang et al., 2017a,b).

Over the last two decades, the field of bone regeneration advanced by the introduction of advanced medical technology and surgical procedures (Rullo et al., 2013), bone morphogenetic proteins and osteoinduction (Urist, 1965; Carragee et al., 2011; Martin et al., 2015) and by the application of research results from the mesenchymal stem cell field (Cancedda et al., 2003; Bianco et al., 2008; Mehrkens et al., 2012; Brennan et al., 2014; Paino et al., 2017) or the neural-crest derived stem cell field (d’Aquino et al., 2011; Spina et al., 2016; Gazarian and Ramírez-García, 2017). In exception of the application of more advanced medical technology or surgical procedures these strategies, however, are costly and associated with increased risks for the patient (Carragee et al., 2011). For the application of bone morphogenetic proteins and stem cells, a carrier system is needed. Ideally, such a delivery system is osteoconductive. In terms of osteoconductive microarchitecture it was shown that bone substitutes containing concave pits induce significantly more bone tissue formation than smooth surfaces (Graziano et al., 2007) and that bone formation benefits from concavities on the surface of calcium phosphate based bone substitutes (Ripamonti, 2017). Another important microarchitectural feature studied for a long time was the optimal pore diameter. As a result of these studies a bone substitute pore diameter of 0.3–0.5 mm has long been regarded as the optimal size for osteoconduction, enabling efficient Haversian-type (Kuboki et al., 2001) and trabecular (LeGeros, 2002) bone formation. There is also the suggestion that bone substitutes with pore sizes in excess of 0.4 mm are less conducive to new bone formation as evidenced by the accumulation of adipocytes and bone marrow (Tsuruga et al., 1997) and reduced mechanical properties (Kuboki et al., 2001). Optimal pore dimension of 0.2–0.5 mm were also supported by several *in vitro* studies (reviewed in Perez and Mestres, 2016). Studies that are more recent reported on bone ingrowth and the presence of cells in micropores, well below 0.1 mm in diameter (Bernstein et al., 2013; Polak et al., 2013). There is only one *in vivo* study with random pore locations and undefined connections between pores suggesting that bone ingrowth is similar in pores from 0.5 mm up to 1.2 mm (von Doernberg et al., 2006). An upper limit in pore diameter for optimal bone ingrowth has not been determined yet.

**FIGURE 1:**
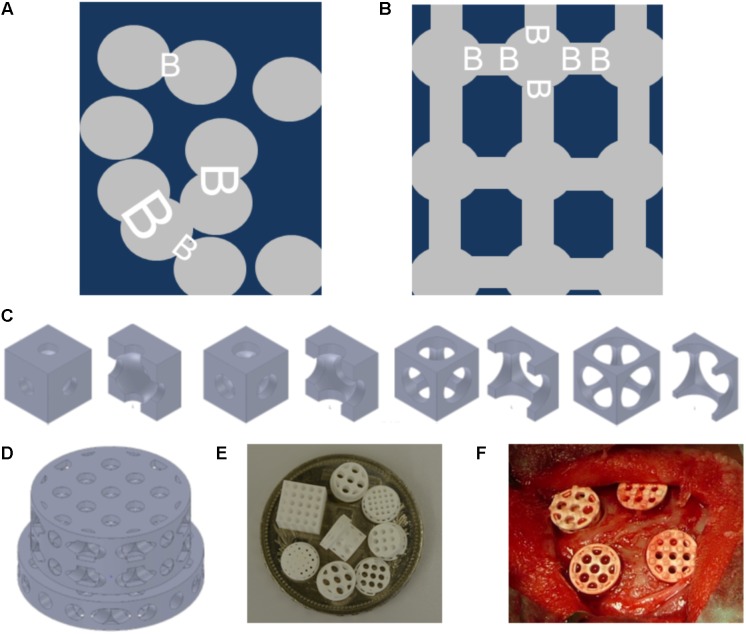
Schematics and examples of bone substitute design, and *in vivo* testing of tri-calcium phosphate based scaffolds. Pore distribution and bottleneck dimension (B) are shown for porosogen based porous scaffolds. Scaffold in dark blue, and pores in gray **(A)**. Pore distribution and bottleneck dimension (B) are shown for scaffolds produced by additive manufacturing. Scaffold in dark blue, and pores in gray **(B)**. Example of unit cells as building block of the scaffolds with fixed cube and pore dimension but increase in bottleneck diameter from left to right. The unit cells views are provided in pairs: left: view on the unit cell, right: view on the halved unit cell **(C)**. Design of one scaffold from the library **(D)**. Scaffolds diverse in pore size and bottleneck dimension are displayed on a Swiss five-franc coin with a diameter of 32 mm **(E)**. Intra operative view of scaffolds placed into four non-critical size defects of 6 mm in diameter created in the calvarial bone of a rabbit **(F)**.

The old dogma of the optimal mean pore size is mainly based on observations using scaffolds with single channels, or randomly distributed pores (**Figure 1A**). The emergence of additive manufacturing has since added a new dimension to the production of scaffolds, where pore size, as well as other microarchitectural constraints such as bottleneck dimensions can be accurately defined (**Figures 1B,C**). The term bottleneck dimension in this context is defined as the uniform diameter of the connections between pores and can be exactly tuned by additive manufacturing. In random pore distribution processes, however, the term percolation diameter was introduced (Ashworth et al., 2015) defined as the diameter of the largest tracer sphere able to move through a scaffold of interconnected pores and reflects the smallest diameter of a single connection in an interconnected pore system.

The effectiveness of 3D printing as an additive manufacturing technique in regenerative medicine, particularly bone tissue engineering, has been well reviewed (Hutmacher, 2000; Jariwala et al., 2015). Using this technology to define bone substitute microarchitecture is, however, still in its infancy (Seitz et al., 2005; Carrel et al., 2016), although the production of fine open structures composed of calcium phosphates is now possible using for example lithography-based additive manufacturing (CeraFab 7500, Lithoz, Vienna, Austria). The aim of this project was to design and produce a library of tricalcium phosphate-based scaffolds with defined pore sizes and bottleneck dimensions using lithography-based additive manufacturing, and to identify the most osteoconductive microarchitecture based on its potential to support defect bridging and new bone formation *in vivo*.

## Materials and Methods

### Design and Fabrication of Scaffolds

We used the computer aided design software tool SolidWorks (Dassault Systèmes SolidWorks Corporation, Waltham, MA, United States) to design a library of 20 different stepped scaffolds with a diameter of 6 mm in the lower three levels, and 7.5 mm in the upper level as previously reported (de Wild et al., 2013) and illustrated in **Figures 1C,D**. The unit cells (**Figure 1C**) to design the scaffolds are cubes of 1.0–2.0 mm length. We have chosen this design and adjusted it to the needs of the material and production methodology, since it resembles the design with the highest mechanical performance and high anisotropy, as previously reported for titanium scaffolds (de Wild et al., 2016, 2018; Rüegg et al., 2017). In the center of each unit cell a pore of 0.5–1.7 mm is located. The pore of each unit cell is connected to all six sides of the cube with a central located cylinder with a diameter between 0.5 and 1.5 mm. The latter diameter is equal to the bottleneck diameter. By the assembly of unit cells to form the scaffold the cylinders are connected and form an open path throughout the entire scaffold. All these parameters from scaffolds of our library are listed in **Table 1**. The fabrication of tri-calcium phosphate based scaffolds was performed using a CeraFab 7500 (Lithoz, Vienna, Austria). We used LithaBone TCP 200 [Ca_3_(PO_4_)_2_] as photosensitive slurry, consisting of tri-calcium phosphate powder of particle size in the range of 5–30 μm, and other undisclosed components like acrylate-based monomer, organic solvent, light absorber and photoinitiator. The CeraFab 7500 (Lithoz, Vienna, Austria) was used to solidify the slurry in a layer-by-layer fashion resulting in a green part with a resolution of 25 μm in layer thickness, and 50 μm in the *x/y*-plane. In the green part, the TCP particles are hold together by the polymer. Following its production, the green parts were removed from the building platform, cleaned from undetached slurry, and underwent a thermal treatment process to remove the solvent, to decompose the polymeric binder, and to sinter (densify) the samples. The program for thermal treatment was provided by the manufacturer, and included a final sintering step of 3 h at 1100°C.

**Table 1:** Structural values for the different scaffolds.

Acronym	Length of unit cell (mm)	Pore diameter (mm)	Bottleneck diameter (mm)	Porosity (Vol %)	Maximal transparency (surface %)	Surface area per Vol (1/mm)
C_10_5_5	1.0	0.5	0.5	35.95	19.64	1.020
C_10_07_05	1.0	0.7	0.5	47.40	19.64	1.774
C_10_07_07	1.0	0.7	0.7	52.58	38.48	1.207
C_13_10_05	1.3	1.0	0.5	47.40	11.61	1.537
C_13_10_07	1.3	1.0	0.7	39.59	22.77	1.279
C_13_10_10	1.3	1.0	1.0	56.00	46.47	0.571
C_15_12_05	1.5	1.2	0.5	32.04	8.72	1.410
C_15_12_07	1.5	1.2	0.7	37.06	17.10	1.242
C_15_12_10	1.5	1.2	1.0	47.47	34.90	0.781
C_15_12_12	1.5	1.2	1.2	56,96	50.26	0.334
C_18_15_12	1.8	1.5	1.2	47.46	34.90	0.630
C_20_17_05	2.0	1.7	0.5	35.64	2.42	1.164
C_20_17_07	2.0	1.7	0.7	36.46	9.62	1.093
C_20_17_12	2.0	1.7	1.2	44.87	28.27	0.711
C_20_17_15	2.0	1.7	1.5	52.03	44.17	0.339

### Scaffold Characterization

The numbers to describe the diverse scaffolds are displayed in **Table 1**. The porosity is the relative free volume describing the ratio of the material free volume inside the unit cell defined by the pore diameter and the diameter of the connections and the volume of the unit cell. The maximal transparency is the material free area in the projection of the unit cell in the spatial direction yielding the maximal value.

### Animal Experiments

All animal procedures were approved by the Animal Ethics Committee of the local authorities (Canton Zurich, 108/2012 and 115/2015) and performed in accordance with the ethics criteria contained in the bylaws of the Institutional Animal Care and Use Committee. After the acclimatization period, implants were inserted into calvarial defects of 40 rabbits (female, 26-week-old, New Zealand white rabbit), and bone regeneration determined after 4 weeks as previously described (Karfeld-Sulzer et al., 2014). In brief, animals were anesthetized by injection of 65 mg/kg ketamine and 4 mg/kg xylazine and further anesthetized with isofluoran/O_2_. The surgical area was disinfected and an incision was made from the nasal bone to the midsagittal crest. Next, the soft tissues were reflected and the periosteum was elevated from the site. In the area of the right and left parietal and frontal bones, four evenly distributed 6 mm diameter craniotomy defects were prepared with a trephine bur under copious irrigation with sterile saline. For the completion of the defect a rose burr (5 mm) was used to preserve the dura. Before implant placement, bone debris were removed by flushing with saline. Each of the animals received four different treatment modalities. The treatment modalities were assigned at random for the first animal and thereafter cyclic permuted clockwise for the next three animals. Sample size was determined by power analysis.

### Histomorphometry

The evaluation of all implants was performed from the MMA-embedded middle section using image analysis software (Image-Pro Plus^®^; Media Cybernetic, Silver Springs, MD, United States). The area of interest (AOI) was defined by the 6 mm defect dimension and the height of the implant, corrected for differences in height between groups of different pore dimension. We determined the area of new bone in the AOI as percent of bone and bony integrated scaffold in the AOI (bony area, %). For the empty control value, the average corrected area occupied by all scaffolds was taken into account.

### Bone Bridging

The determination of bone bridging was performed as previously reported (Kruse et al., 2011; Schmidlin et al., 2013). In brief, areas with bone tissue were projected onto the *x*-axis. Next, the stretches of the *x*-axis where bone formation had occurred at any level were summed up and related to the defect width of 6 mm. Bone bridging is given in percentage of the defect width (6 mm) where bone formation had occurred.

### Statistical Analysis

The primary analysis unit was the animal. For all parameters tested, treatment modalities were compared with a Kruskal–Wallis test, followed by Mann–Whitney signed rank test for independent data (IBM SPSS v.23). Significance was set at *P* < 0.05. Values are reported as either mean ± standard error, or displayed in box-plots ranging from the 25th (lower quartile) to the 75th (upper quartile) percentile including the median and whiskers showing the minimum and maximum values.

## Results

### Scaffolds With Pores Between 0.7 and 1.2 mm Are Optimal for Calvarial Bone Healing

A total of 20 different scaffolds were prepared using the computer aided design software tool SolidWorks (Dassault Systèmes SolidWorks Corporation, Waltham, MA, United States), and their potential to support new bone growth assessed in a rabbit calvarial defect model (de Wild et al., 2013) (**Figure 1E**). Scaffolds were generated from the tri-calcium phosphate substrate Lithabone^TM^ (Lithoz, Vienna, Austria) using the lithography-based additive manufacturing machine (CeraFab 7500, Lithoz, Vienna, Austria). Five scaffold designs failed mechanically during production, or during the *in vivo* testing stage, and are therefore not reported here. Following the removal of the photoactive binder, and sintering to increase mechanical stability all scaffolds exhibited a smooth surface with micropores of 2–4 μm (Chen et al. accepted Tissue Engineering). The so produced scaffolds, sterilized during the sintering procedure (**Figure 1E**) were transferred to the operation theater in a sterile fashion, implanted within the calvarial defects (**Figure 1F**), and bone formation assessed after 4 weeks.

**FIGURE 2:**
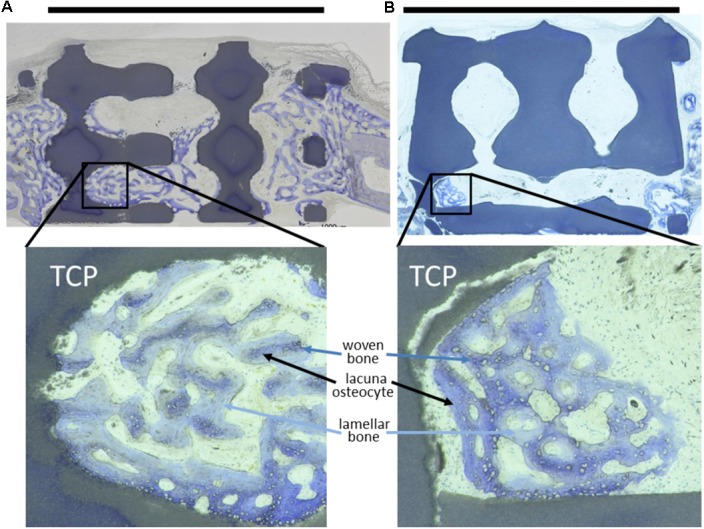
Toluidine-blue stained MMA sections of middle sections from scaffold treated calvarial bone defects. Upon a healing period of 4 weeks, the scaffolds from tricalcium phosphate (TCP) are visible as blackish or dark blue areas. Bone ingrowth was most advanced in additively manufactured scaffolds with a pore diameter of 1.2 mm and bottleneck diameter of 1.0 mm **(A)**. In scaffolds with a pore diameter of 1.5 mm and bottleneck diameter of 1.0 mm **(B)** bone growth did not extend far into the scaffolds. A scale bar, placed on top of the histologies is provided to show the 6 mm defect margins. In the lower panel, 2.5-fold higher magnifications are shown from the area marked in **(A,B)** to visualize bone tissue formation. The light blue-stained bone tissue is lamellar bone (also new, but later formed) on the initial woven bone structures stained dark blue and purple. Lacunae from osteocytes are visible.

Histological analysis of methyl methacrylate (MMA)-embedded tissue sections revealed predominantly woven bone formation in and around the scaffolds indicating good biocompatibility by new bone formation in close proximity to the scaffold material (**Figure 2**). Evaluation of osteoconduction was based on the level of bony bridging and bony regenerated area as determined by toluidine-blue staining. As compared to untreated empty defects, defects containing scaffolds with pore diameters of 1.2 mm and below performed better in terms of bony bridging and/or bony regeneration than scaffolds with a pore diameter of 1.5 mm or 1.7 mm.

**FIGURE 3:**
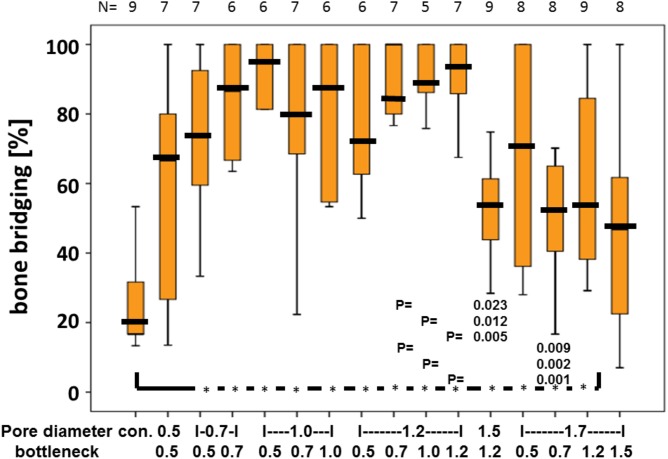
Percentage of bony bridging of the defect in relation to pore and bottleneck dimensions. In comparison to the empty control, scaffolds with pore diameters from 0.7 to 1.7 mm and bottlenecks below 1.5 mm perform significantly better. Scaffolds with pores of 1.2 mm and bottlenecks between 0.7 and 1.2 mm perform significantly better than scaffolds with a pore diameter of 1.5 mm and bottleneck of 1.2 mm and pore diameter of 1.7 mm and bottleneck of 0.7 mm. Results of each group are displayed as box-plots ranging from the 25th (lower quartile) to the 75th (upper quartile) percentile including the median as black bars and whiskers showing the minimum and maximum values. The number of samples for each group (N) is displayed on top of the figure.

Quantitative analysis of the middle sections further confirmed that the percentage of bony bridging compared to untreated control defects was significantly greater in animals treated with scaffolds with pore diameters of 0.7, 1.0, 1.2, 1.5, and 1.7 mm. Furthermore, scaffolds with pore sizes of 1.0 mm and bottlenecks of 0.7 and 1.0 mm and pore size of 1.2 mm and bottlenecks of 0.7, 1.0, and 1.2 mm proved significantly better as compared to scaffolds with pores sizes of 1.5 and 1.7 mm, and bottlenecks of 0.7 and 1.2 mm, respectively (**Figure 3**). Pore diameters of 1.5 and 1.7 mm were therefore considered the least beneficial for bony bridging.

The optimal pore diameter and bottleneck dimension for an osteoconductive scaffold is between 0.7 and 1.2 mm and below 1.5 mm.

**FIGURE 4:**
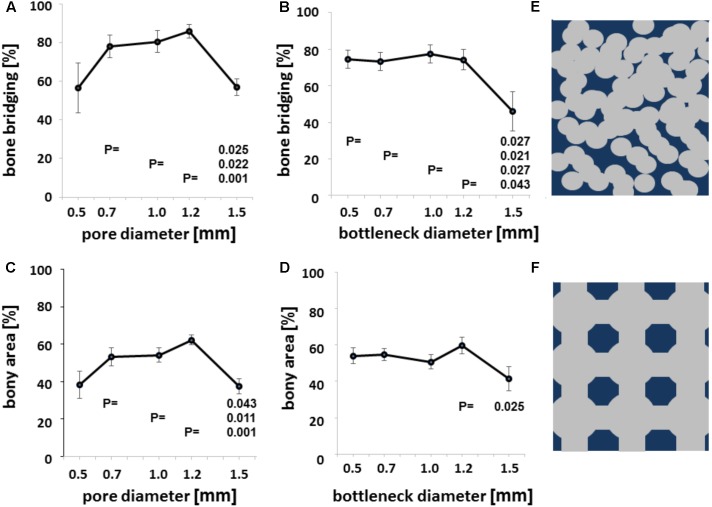
Microarchitecture and osteoconduction. Bony bridging in relation to pore diameter of all groups **(A)**. Bony bridging and bottleneck diameter. Bony bridging in relation to bottleneck diameter of all groups **(B)**. Bony regenerated area and pore diameter. Bony regenerated area in relation to bottleneck diameter of all groups **(C)**. Bony regenerated area and bottleneck diameter. Bony regenerated area in relation to bottleneck diameter of all groups **(D)**. All graphs display the means and the standard error of the means. *P*-values are displayed. Schematic drawing of conventional scaffold based on random distributed 0.5 mm diameter pores. Scaffold in dark blue and pores in gray **(E)**. Schematic drawing of additively manufactured scaffold based on 1.2 mm pores and bottlenecks of 0.7 mm Scaffold in dark blue and pores in gray **(F)**.

We next evaluated bony bridging and bony regenerated area dependency, grouped by pore diameter and bottleneck dimension. We found bony bridging of the defect to be significantly more complete in scaffolds with pores of 0.7 to 1.2 mm in diameter as compared to a pore diameter of 1.5 mm or 1.7 mm (**Figure 4A**). For the percentage of bony regenerated area in the defect, a pore diameter of 0.7–1.2 mm was significantly superior to a pore diameter of 1.5 mm (**Figure 4C**).

The percentage of bony bridging of the defect and the percentage of bony regenerated area of the defect of scaffolds with 0.5 mm pores were always in the range of scaffolds with 1.5 mm diameter pores and below scaffolds with pores of 0.7, 1.0, or 1.2 mm in diameter. Therefore, optimal pore diameter for both measures of osteoconductivity lies between 0.7 and 1.2 mm. If the bottleneck between pores is between 0.5 and 1.2 mm, bony bridging is significantly higher than for bottlenecks of 1.5 mm in diameter (**Figure 4B**). For bony regenerated area, only a bottleneck dimension of 1.2 mm was significant higher than one of 1.5 mm (**Figure 4D**). Grouping defect bridging according to porosity or transparency (**Table 1**) did not yield in any significant differences. The same applies to surface area per volume. Therefore, in this library of scaffolds from the identical material and identical surface structure pore diameter and bottleneck dimension were the key parameters of the microarchitecture to affect bony bridging and bony regeneration as measures for osteoconduction.

## Discussion

In the current report, we generated a structurally diverse library of tri-calcium phosphate based scaffolds using 3D printing, and assessed their potential to influence bone formation *in vivo*. The osteoconductive capacity of bone implants is reliant on a complex interplay between the material type, surface and microarchitecture, which ultimately determines the efficiency of new bone formation and its vascularization. Here, we kept the material and surface characteristics constant and varied only the microarchitecture. The primary focus of our study was to challenge the long-held belief that optimal osteoconduction is achieved using bone substitutes with pore diameters of 0.3–0.5 mm (Tsuruga et al., 1997; Langer and Vacanti, 1999; Hutmacher, 2000; Galois and Mainard, 2004; Hollister, 2005; Murphy et al., 2010; Hollister and Murphy, 2011; Henkel et al., 2013) thereby opening up new possibilities for the improvement of scaffold microarchitecture design.

The results of our study demonstrated osteoconduction was significantly improved in bone substitutes with a pore diameter of 0.7–1.2 mm — an increase of up to fourfold above what the majority of published studies and reviews recommend. These findings are likely to pave the way for future developments in scaffold design, leading to the generation of bone substitutes with a more osteoconductive microarchitecture, and improved bone-regenerative capability (**Figures 4E,F**). Importantly, a pore size of 1.5 mm or greater had a detrimental effect on the bone bridging capabilities of the tri-calcium phosphate scaffolds used in this study; a notable diagnostic feature of non-unions. Given the clinical and economic burden of treating non-unions (Zura et al., 2016), there is an obvious need to develop bone substitutes with high osteoconductive properties.

Moreover, these data corroborate our previous observations on the influence of pore size on osteoconduction of titanium scaffolds (de Wild et al., 2013, 2016, 2018). Our results are in line with an *in vivo* sheep study reporting on drill hole defects in cancellous bone where scaffolds with random distributed pore sizes of 0.15, 0.26, 0.51, and 1.22 mm but undefined pore location and bottleneck dimension or percolation were tested (von Doernberg et al., 2006). In terms of bone regeneration, they did not see huge differences. It should be noted, however, that due to technical restraints of our additive manufacturing process, generating bone substitutes with pore sizes below 0.5 mm was not technically possible— n obvious limitation of this system. Importantly, a pore size of 1.5 or 1.7 mm or a bottleneck of 1.5 mm had a detrimental effect on the bone bridging and bony regeneration capabilities of the tri-calcium phosphate scaffolds used in our study. This sets a so far unknown upper limit to osteoconductive pore sizes at 1.2 mm and below 1.5 mm.

For polycaprolactone based scaffolds, a more permeable scaffold with regular architecture performed best for *in vivo* bone regeneration (Mitsak et al., 2011). The microarchitecture we have tested here is not only permeable but also transparent from each plane of the cube of the unit cell throughout the entire scaffold, since all empty cylinders connecting the pores are aligned. As listed in **Table 1**, the transparency of unit cells 1.5 mm in length and a pore diameter of 1.2 mm increases with the bottleneck diameter (0.5–1.2 mm) from 8.72 to 50.26%. Bony bridging for those scaffolds follow the same trend, but fail to differ significantly. The same applies to porosity, which also increases from 32.04 to 56.96%. Scaffolds with pores of 1.5 mm perform significantly worse in terms of bony bridging, despite the fact that with a transparency of 34.90% and a porosity of 47.46% both characteristics are more at the higher end. Moreover, the surface area per volume of scaffolds derived from the unit cell of 1.5 mm in length and a pore diameter of 1.2 mm decreases with the increase in bottleneck diameter (0.5–1.2 mm from 1.41 to 0.33 1/mm (**Table 1**). Therefore, bony bridging and surface area follow opposite trends. Taken together, with our microarchitecture bony bridging follows permeability and porosity in groups derived from the same unit cell. The most important factor, however, appears to be pore diameter. To fully understand all these relations, additional research with more designs and additional model systems are needed.

Interestingly, it was proposed that in the initial weeks bone regeneration depends mainly on material aspects and that design aspects come in play only at later stages (Kommareddy et al., 2010; Tamjid et al., 2013). This is true for *in vitro* situations. *In vivo*, however, we show that with a constant material and surface structure, osteoconduction even during the first 4 weeks depends heavily on pore size and bottleneck dimension and therefore on microarchitectural features. That *in vitro* and *in vivo* results on bone tissue engineering approaches can contradict each other has been noted by others as well (Karageorgiou and Kaplan, 2005).

Autologous bone is still the bone substitute material of choice for treating critical size defects (De Long et al., 2007). Since the porosity of trabecular bone is between 0.2 and 0.4 mm (LeGeros, 2002), it was reasonable to assume that scaffolds with comparable pore dimensions would provide a more physiologically relevant bone substitute. In living bone, however, the microarchitecture reflects the local mechanical needs (Keaveny et al., 2001) and thus, no evolutionary pressure exists on osteoconduction in terms of bone ingrowth into 3D-structures. One can speculate that the optimal pore and bottleneck dimensions derived from this study reflect the balance between the positive interactions of directionally growing bone tissue with the scaffold as guiding cue, and the restrictions imposed by the scaffold on directional bone growth. Clearly, this balance is tipped in favor of the scaffold’s positive effects, as made evident by the significant improvement in bony bridging when using bone substitutes with pore sizes between 0.7 and 1.2 mm, and bottlenecks between 0.5 and 1.2 mm instead of pores and bottlenecks of 1.5 mm and more (**Figure 3**).

Our scaffolds consisted of tri-calcium phosphate, which is often used for scaffolds in bone tissue engineering as it degrades faster than native hydroxyapatite, whilst remaining biodegradable even after sintering at temperatures above 1,100°C (Goto et al., 2001). However, in the current study, scaffold biodegradation was not taken into consideration based on the fact that it takes several months for tri-calcium phosphate to completely be removed from the defect site (Walsh et al., 2008).

Additive manufacturing of free form scaffolds alleviated some of the constraints of extrusion-based techniques, such as the filament dimension ruling the pore dimension in the *z*-axis and the mechanics of the filament the pore dimension in the *x*- to *y*-axis. In the past, porogens were considered a necessary component of the porous bone substitute production process, but led to random pore distribution, uncontrollable bottleneck dimensions, and restricted research of scaffolds microarchitecture (**Figure 4E**). We envisage that the use of additively manufactured bone substitutes with pore diameters in the range of 0.7–1.2 mm, and a bottleneck dimension of 0.5–1.2 mm, offer the best solution achieving optimal bone regeneration, and have the potential to revolutionize the way we treat bone defects.

## Conclusion

The microarchitecture of bone substitutes based on pores and bottlenecks is most osteoconductive with pore diameters between 0.7 and 1.2 mm and bottlenecks between 0.5 and 1.2 mm. Pores and bottlenecks of 1.5 mm and beyond are detrimental for osteoconduction. In order to generate such microarchitecture, additive manufacturing is likely to become a central player, enabling the production of reproducible osteoconductive microarchitectures, which can be adjusted according to mechanical needs. Furthermore, additive manufacturing will be an invaluable tool in developing strategies geared toward personalized treatment, where the generation of scaffolds with patient specific bone defect dimensions is highly desirable.

## Author Contributions

FW designed the experiments. CG and FW performed the experiments, analyzed the data, and wrote the manuscript.

## Conflict of Interest Statement

The University Zurich has filed a patent on the microarchitecture of osteoconductive bone substitute with FW listed as sole inventor. The remaining author declares that the research was conducted in the absence of any commercial or financial relationships that could be construed as a potential conflict of interest. The reviewer VT and handling Editor declared their shared affiliation.
